# Newly Emerging Strategies in Antiviral Drug Discovery: Dedicated to Prof. Dr. Erik De Clercq on Occasion of His 80th Anniversary

**DOI:** 10.3390/molecules27030850

**Published:** 2022-01-27

**Authors:** Shujing Xu, Dang Ding, Xujie Zhang, Lin Sun, Dongwei Kang, Boshi Huang, Xinyong Liu, Peng Zhan

**Affiliations:** 1 Key Laboratory of Chemical Biology (Ministry of Education), Department of Medicinal Chemistry, School of Pharmaceutical Sciences, Shandong University, 44 West Culture Road, Jinan 250012, China; xu17854111942@163.com (S.X.); DangDing0302@163.com (D.D.); sduzxj1998@163.com (X.Z.); sunlin6018@163.com (L.S.); kangdongwei@126.com (D.K.); 2 Department of Medicinal Chemistry, School of Pharmacy, Virginia Commonwealth University, 800 E Leigh Street, Richmond, VA 23298, USA; bhuang2@vcu.edu

**Keywords:** viruses, antiviral drugs, medicinal chemistry strategies, drug design

## Abstract

Viral infections pose a persistent threat to human health. The relentless epidemic of severe acute respiratory syndrome coronavirus 2 (SARS-CoV-2) has become a global health problem, with millions of infections and fatalities so far. Traditional approaches such as random screening and optimization of lead compounds by organic synthesis have become extremely resource- and time-consuming. Various modern innovative methods or integrated paradigms are now being applied to drug discovery for significant resistance in order to simplify the drug process. This review provides an overview of newly emerging antiviral strategies, including proteolysis targeting chimera (PROTAC), ribonuclease targeting chimera (RIBOTAC), targeted covalent inhibitors, topology-matching design and antiviral drug delivery system. This article is dedicated to Prof. Dr. Erik De Clercq, an internationally renowned expert in the antiviral drug research field, on the occasion of his 80th anniversary.

## 1. Introduction

Emerging infectious diseases have threatened humanity throughout history [1]. Infectious diseases have accounted for 20% of global mortality, and viral diseases have caused 70% of these deaths [2,3]. During the last century, viruses were responsible for massive numbers of deaths; for example, smallpox killed nearly 400 million people during the 20th century [4], and influenza was accountable for roughly 100 million deaths during the major outbreak in 1918–1919 known as the Spanish flu [5]. The epidemic of human immunodeficiency virus (HIV) has caused 35 million deaths since it was first recognized in 1981 [6,7]. The coronavirus disease 2019 (COVID-19) pandemic caused by severe acute respiratory syndrome coronavirus 2 (SARS-CoV-2) was also considered “among the deadliest pandemics of the past century” [8,9], and as of January 25, 2022, confirmed cases of COVID-19 reached 352 million with 5.60 million deaths (https://covid19.who.int, accessed on 26 January 2022). Therefore, viral infection has become a major public safety and health problem that seriously threatens human health. Currently, the prevention and treatment of human viral infections mainly rely on combination drugs and vaccines [2,10]. However, existing antiviral drugs are facing the dilemma of increasing drug-resistant strains [11], while vaccines often do not work against mutated or novel viruses. Therefore, it is essential to explore innovative strategies in antiviral drug discovery.

**Figure 1:**
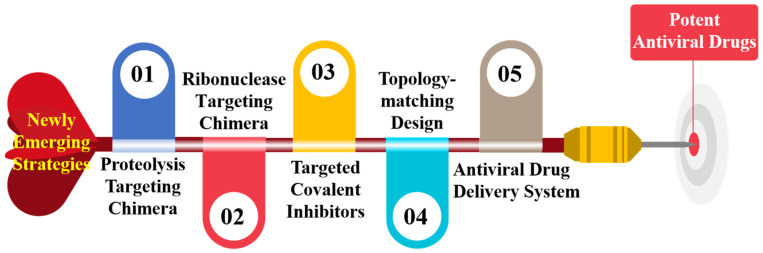
Newly emerging strategies in antiviral drug discovery.

The traditional approach of random screening and subsequent optimization of lead compounds by the systematic chemical synthesis is highly resource- and time-consuming [12]. Thus, more efficient and faster strategies to shorten and facilitate the discovery process will be beneficial. In recent years, several strategies have been employed to discover novel antiviral agents with new scaffolds and better resistance profiles, including proteolysis targeting chimera (PROTAC), ribonuclease targeting chimera (RIBOTAC), targeted covalent inhibitors, topology-matching design and antiviral drug delivery systems (Figure 1). Implementation of these newly emerging medicinal chemistry strategies described herein are expected to help to find potent antiviral drugs that effectively respond to the current and future threats posed by emerging and re-emerging viral pandemics.

## 2. Proteolysis Targeting Chimera (PROTAC)

PROTACs have become an emerging drug discovery paradigm to target proteins through promoting and realizing the degradation of target proteins via the ubiquitin–proteasome system (UPS) [13]. PROTACs are hetero-bifunctional molecules consisting of a ligand for the protein of interest (POI), an E3 ubiquitin ligase recruitment ligand and a linker. Bifunctional PROTAC molecules bind to the POI with one end, while the other end binds to an E3 ligase to shorten the distance between them in vivo. The E3 ligase then mediates the transfer of ubiquitin from an E2 enzyme to the POI, and finally the ubiquitylated POI is knocked down by the proteasome [14,15]. Recently, this methodology has been gradually applied to the discovery of antiviral agents.

**Figure 2:**
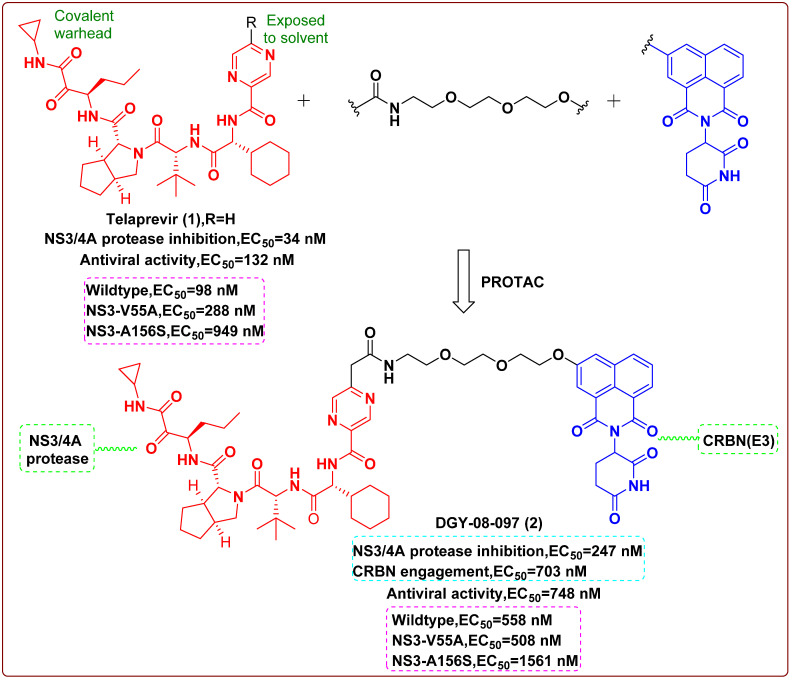
Chemical structures and inhibitory activities of telaprevir (**1**) and the degrader derivative DGY-08-097 (**2**) [16].

In 2019, Yang et al. [16] reported a PROTAC molecule that could degrade the hepatitis C virus (HCV) protease. Telaprevir (**1**), a reversible covalent inhibitor binding to the active site of HCV protease, the cocrystal structure of telaprevir in complex with the viral protease, showed that the solvent-exposed pyrazine ring could be derivatized with different linkers conjugated to chemical binders of cereblon (CRBN), the substrate receptor for the CUL4–RBX1–DDB1–CRBN E3 ubiquitin ligase complex (CRL4^CRBN^). Therefore, telaprevir could serve as a protein–ligand, being conjugated to ligands that recruit the CRL4^CRBN^ ligase complex, producing compounds that could both inhibit and induce the degradation of the HCV NS3/4A protease. The CRBN-binding moieties of the PROTAC molecules were derived from lenalidomide, pomalidomide or a tricyclic imide moiety. An optimized one, DGY-08-097 (**2**, Figure 2), effectively inhibited HCV in a cellular infection model (EC_50_ = 748 nM), which proved that the degradation of protein was helpful to its antiviral activity. Finally, the researchers concluded that these new types of antiviral agents could overcome viral variation and thereby solve drug-resistance to traditional enzymatic inhibitors. The finding confirmed that these small-molecule degraders were less vulnerable to mutations that affected ligand binding and could be employed to inhibit or treat viral variants associated with resistance to traditional inhibitors.

Additionally, PROTACs also have the advantages of low dosage and toxicity, as well as high selectivity. Therefore, this study demonstrated that there is a tremendous opportunity to apply targeted protein degradation as a complementary methodology to accelerate the discovery of antiviral drugs.

## 3. Ribonuclease Targeting Chimera (RIBOTAC)

RIBOTAC is a new strategy for RNA degradation. RIBOTAC includes an RNA-binding small molecule and a ribonuclease (RNase) L-recruiting module aiming to degrade the viral genome [17,18]. RNase L acts in innate immunity and is expressed at minute levels as an inactive monomer in all cells, which is activated and dimerized during viral infection with inherent substrate specificity [19]. RIBOTACs locally recruit RNase L to the expected target to achieve the effect of selective cleavage.

**Figure 3:**
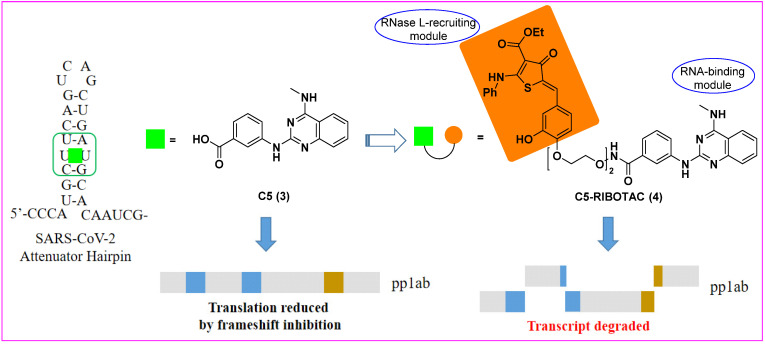
Chemical structures of C5 (**3**) and C5-RIBOTAC (**4**, the heterocyclic recruiter of RNase L is shown in orange) and schematic of C5-RIBOTAC degradation of the SARS-CoV-2 RNA [20].

In 2020, Haniff et al. [20] designed multiple bioactive small molecules targeting a functional structure within the RNA genome of SARS-CoV-2. An analysis of RNA genome structure afforded a modified model of the SARS-CoV-2 frameshifting element (FSE), especially its attenuator hairpin, which controlled the translation of pp1a and pp1ab polyproteins that were essential for viral replication and pathogenesis. Using Absorb Array and luciferase reporter-based cellular assays, they identified a drug-like small molecule (C5, **3**) that selectively bound to and stabilized the revised attenuator hairpin structure of FSE with a *K*_d_ of 11 nM, reducing its frameshifting efficiency in cells. The ligand was further elaborated into an RNA degrader (C5-RIBOTAC, **4**) to recruit a cellular ribonuclease to destroy the viral genome that validated direct target engagement and enhanced antiviral potency via targeted degradation of the viral RNA (Figure 3). Eventually, the RIBOTAC-based lead optimization strategy enhanced the antiviral activity of the lead compound at least 10-fold. Collectively, this study suggested that RIBOTAC could be a new direction towards the discovery of viral RNA genome-targeting agents.

## 4. Targeted Covalent Inhibitors

The development of structural biology and bioinformatics has greatly promoted the rational design of targeted covalent inhibitors (TCIs). Covalent inhibitors can interact with specific target proteins to form covalent bonds that result in changes in the conformation of proteins, thus interfering with the normal function of the protein [21]. The covalent binding with the target can be divided into two related but discontinuous processes: (i) the inhibitor reversibly binds to the target, making the functional groups on the weak electrophilic ligands adjacent to the specific nucleophilic residues on the protein; (ii) the ligand reacts with the functional groups involved in the protein to form a covalent bond [22,23]. In recent years, TCIs have received growing attention from the antiviral field due to their significant advances in terms of efficacy and selectivity. 

**Figure 4:**
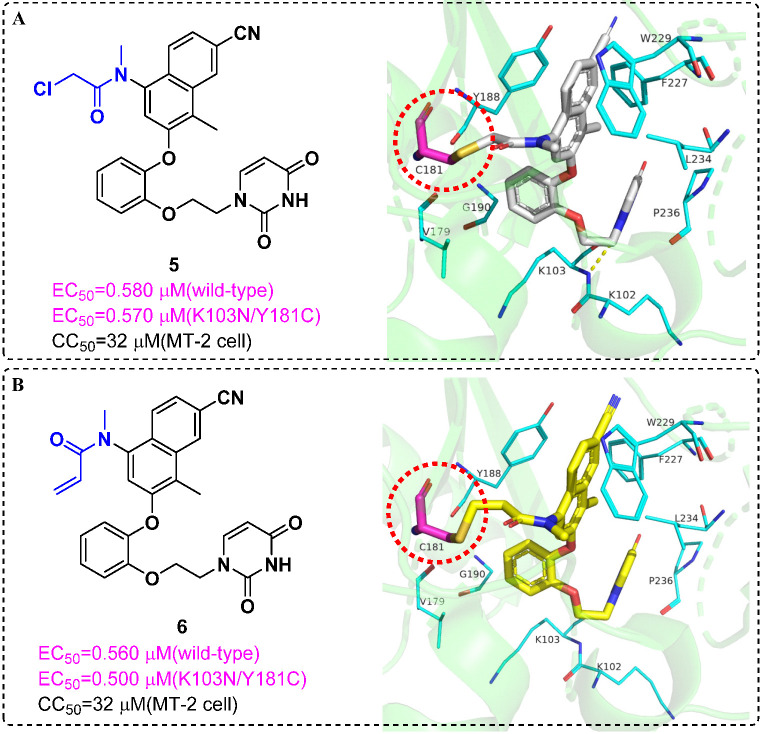
(**A**) Chemical structure of **5** and crystal structures of Y181C RT in complex with **5** (PDB code: 5VQX); **5** forms a covalent bond with the sulfhydryl group of Cys181. (**B**) The same as A but with Y181C:**6** (PDB code: 5VQV) [24]. The figures were generated by PyMol.

Resistance related to the Tyr181Cys (Y181C) mutation in HIV-1 reverse transcriptase (RT) is one of the main obstacles for the development of nonnucleoside RT inhibitors (NNRTIs). In 2017, Chan et al. [24] reported covalent inhibitors of Y181C RT that could completely knock out activity of the resistant mutant. Enzyme inhibition kinetics, mass spectrometry, protein crystallography and antiviral activity detection provided compelling evidence for covalent modification of Cys181. Success was obtained for the chloromethylamide **5** and the acrylamide **6**, and they could form covalent bonds with the sulfhydryl group of Cys181; it may be possible to dose them less frequently than noncovalent inhibitors (Figure 4). It was the first time that an irreversible covalent inhibition strategy was successfully applied to HIV-1 RT; diversity-oriented warhead selection made it possible to systematically explore chemical space.

**Figure 5:**
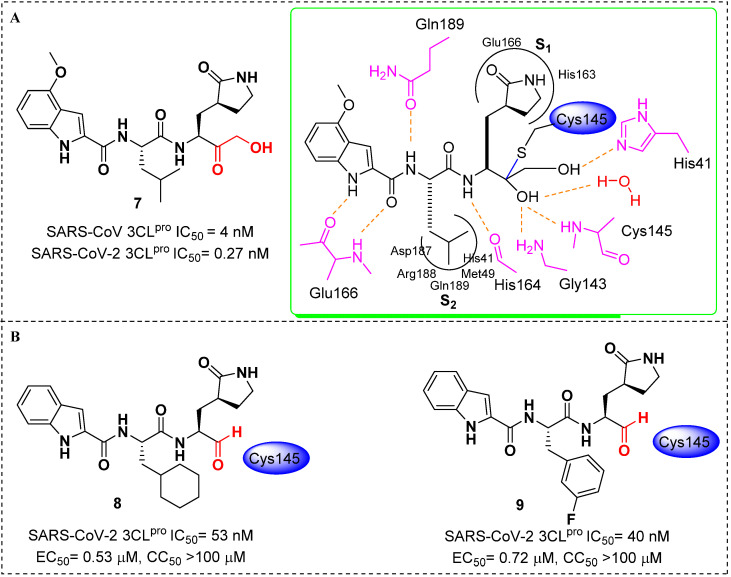
(**A**) Chemical structure of **7** and the schematic rendering of the active site with dashed lines represented as hydrogen bonds with key residues and curved lines to show S1 and S2 binding pockets [25]; (**B**) chemical structures of **8** and **9**; inhibitory activities against SARS-CoV-2 3CL^Pro^(IC_50_) and in vitro inhibition of 3CL^Pro^(EC_50_) [26].

In 2020, Hoffman et al. [25] reported the discovery and characterization of a potent ketone-based covalent inhibitor of SARS-CoV-2 coronavirus 3CL protease (3CL^pro^). 3CL^pro^, as the main protease, is critical for mediating viral replication and transcription. The hydroxymethylketone derivative **7** exhibited potent SARS-CoV inhibition in 3CL^pro^ and antiviral assays. Cocrystal structures of **7** in complex with 3CL^pro^ of SARS-CoV-2 confirmed that the warhead hydroxymethylketone carbonyl carbon of **7** formed a covalent bond to the sulfur of the Cys145 in 3CL^pro^ active-site, producing a tetrahedral carbinol complex. This carbinol hydroxyl formed hydrogen bonds with the backbone NH of Cys145 and with the amide NH of Gly143 via a bridging water molecule. Another key active-site interaction was the hydrogen bond between the primary alcohol moiety of **7** and the catalytic His41 (Figure 5A). Additionally, **7** displayed acceptable solubility, stability in plasma and low in vitro and in vivo clearances, which were suitable for further development as an anti-SARS-CoV-2 drug candidate. Moreover, Dai et al. [26] also reported two potent inhibitors (**8** and **9**, Figure 5B) that were covalently bound to Cys145 of 3CL^pro^. Both of them showed good pharmacokinetic properties in vivo, and **8** also exhibited low toxicity, suggesting that these compounds are promising anti-SARS-CoV-2 drug candidates.

## 5. Topology-Matching Design

Influenza A virus (IAV) is an enveloped RNA virus, of which the membrane anchors two viral proteins that regulate interactions between the virion and host cells, namely hemagglutinin (HA) and neuraminidase (NA) [27]. From a topological viewpoint, the virion of IAV is a nanosized particle of about 100 nm with a spiky surface created by the HA and NA [28]. For nano-inhibitors, it is crucial to match the size and topology of the virion in order to achieve competitive binding with the virus/cell interaction.

**Figure 6:**
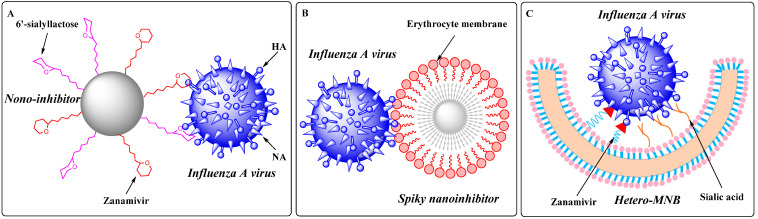
(**A**) Proposed binding patterns between nano-inhibitor and IAV particles [29]; (**B**) proposed binding patterns between spiky nanoparticle-based inhibitor and IAV particles [30]; (**C**) proposed binding patterns between IAV and the heteromultivalent nanobowl (Hetero-MNB), where sialic acid and zanamivir bind to HA and NA, respectively, and the bowl shape facilitating the capping to the surface of the virus particle [31].

In 2020, Nie et al. [29] demonstrated the concept of “topology-matching design” for virus inhibitors. They designed a nano-inhibitor with a nano-topological structure matching the IAV virions and showed hetero-multivalent inhibitory effects on HA and NA (Figure 6A). The synthesized nano-inhibitor could neutralize the viral particle extracellularly and block its attachment and enter host cells. The virus replication was substantially reduced by six orders of magnitude, reaching more than 99.999% inhibition even after 24 h of infection, which demonstrated that such a nano-inhibitor might be a potent anti-influenza agent. Moreover, they also found a spiky nano-inhibitor with matched topography to IAV virions. Due to the short spikes inserted into the glycoprotein gap of the IAV virion, the binding of the nanostructures with spikes between 5 and 10 nm was substantially better than that of smooth nanoparticles (Figure 6B). In addition, targeting IAV by an erythrocyte membrane (EM) could efficiently prevent IAV virion binding to the cells and inhibit subsequent infection. In a post-infection study, the EM-coated nanostructures could reduce virus replication by more than 99.9% at the cellular nontoxic dosage [30]. 

In 2021, the same group reported heteromultivalent topology-matched nanostructures as effective and broad-spectrum IAV inhibitors. The heteromultivalent binding moieties were transferred to bowl-like nanostructures that matched the spherical surface of the virus, coating the inhibitor surface with a cell-derived membrane as a native source of sialic acids and complementing the cell-derived membrane with zanamivir to increase the IAV–membrane interaction by heteromultivalent binding (Figure 6C). Unlike the traditional homomultivalent inhibitors, the IC_50_ value of the heteromultivalent inhibitors was 32.4 ± 13.7 ug/mL owing to the synergistic multivalent effects and the topology-matched shape. The virus propagation was reduced by more than 99.99% at a dose that did not cause cytotoxicity. Since multiple binding sites have also been identified on the S protein of SARS-CoV-2, it is envisaged that heteromultivalent nanostructures may also be employed in seeking effective SARS-CoV-2 inhibitors [31].

## 6. Antiviral Drug Delivery System

Human serum albumin (HSA) is the most abundant protein in sera (30–50 g/L in human serum), where it primarily functions as a natural transporter of various molecules [32]. As an inherent protein in human blood, it does not exhibit immunogenicity. Non-covalent binding of small molecular drugs to HAS protects them from enzymatic degradation and renal clearance, providing slower clearance and extended half-life in vivo [33]. Thereupon HSA is an ideal drug carrier for targeting delivery and improving the pharmacokinetic profile (half-life extension) of drugs. 

**Figure 7:**
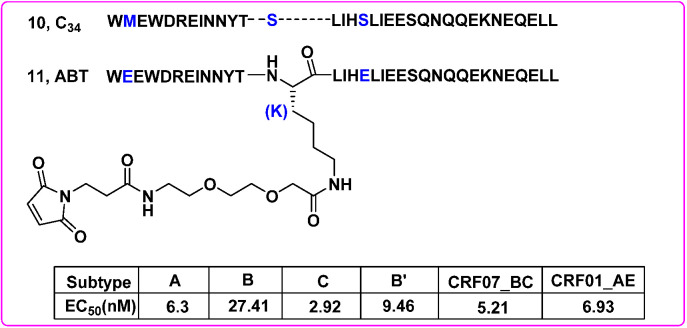
The discovery of ABT and inhibitory potency against a set of HIV-1 subtypes [34].

On 6 June 2018, Albuvirtide (ABT), an HIV fusion inhibitor developed by Frontier Biotechnologies, was approved as a novel anti-HIV drug in China. ABT is a 3-maleimidopropionic acid (MPA)-modified peptide designed with the C34 (**10**) sequence as a template. Among them, the 13th residue serine (S) was replaced by lysine (K), allowing a single MPA modification at this position. The other two non-target binding residues were replaced by glutamic acid (E) to improve solubility, stability and antiviral activity (Figure 7). It was shown that ABT (**11**) irreversibly bound to serum albumin and prolonged its half-life. The chemically modified ABT could form a stable helical structure with the target sequence, effectively blocking the formation of 6-HB (EC_50_ = 0.82 μM) and HIV-1 Env-mediated cell–cell fusion (EC_50_ = 1.27 nM). Notably, it inhibited the entry of various HIV-1 subtypes and variants, including the subtypes A, B and C that predominate the global AIDS epidemics, and subtype B’, CRF07_BC and CRF01_AE recombinants that are currently circulating in China [34] (Figure 7). Furthermore, a phase III clinical trial (TALENT study) demonstrated that the injectable long-acting HIV-1 drug ABT combined with ritonavir-boosted lopinavir (LPV/r) was both safe and effective [35]. However, ABT presents several limitations for its intravenous use only. Even so, the discovery of ABT suggested that targeting HSA was a promising and feasible way to develop long-acting antiviral drugs. 

Cholesterol is abundant in eukaryotic cell membranes. Cholesterol conjugation can spontaneously insert modified nucleic acids and peptides into lipid bilayers and their subsequent uptake by cells [36]. This membrane-targeting strategy can be particularly helpful in enhancing the antiviral efficacy of NA inhibitors because they block the NA enzyme activity on the surface of infected cells, leading to the release and transmission of progeny virus [37].

**Figure 8:**
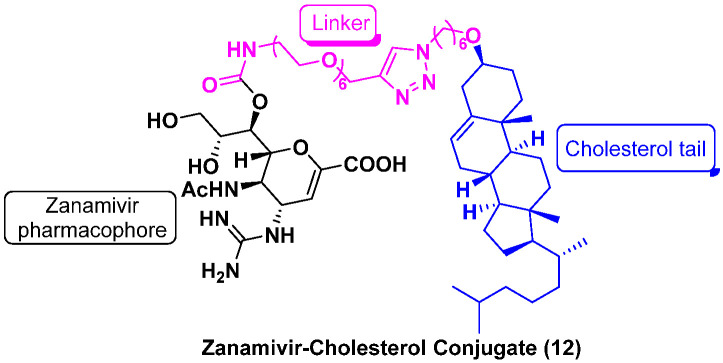
Chemical structure of zanamivir–cholesterol conjugate (**12**) [38].

In 2021, Lv et al. [38] found that the zanamivir (ZNV)–cholesterol conjugate (**12**, Figure 8) was a long acting neuraminidase inhibitor with potent efficacy against drug-resistant influenza viruses (Table 1). Compared with ZNV (t_1/2_ = 0.3 h, in rats), the antiviral efficacy and plasma half-life **12** (t_1/2_ = 7.6 h, in rats) were significantly improved. Single-dose administration of the conjugate protected the mice from lethal challenges of wild-type or mutant H1N1 influenza viruses bearing an oseltamivir (OSV)-resistant H275Y-mutation. Mechanistic studies confirmed that the conjugate targeted the cell membrane and entered the host cells, thereby inhibiting the NA function and progeny virion assembly. Therefore, this study validates cholesterol conjugation as an effective strategy for improving potency and pharmacokinetics of other small-molecule agents.

## 7. Conclusions

Chronic viral infections such as HIV and hepatitis B, as well as the emergence of new viruses such as Ebola and coronaviruses (SARS-CoV, SARS-CoV-2) highlight the need for more innovative strategies to develop better antiviral drugs. In this review, we have briefly described several newly emerging strategies employed for developing antiviral agents, which have been used to discover novel drug entities, to improve anti-drug-resistance profiles and potency. However, these strategies also have some limitations. PROTACs must cross the cell membrane to achieve intracellular protein degradation, but the large molecular weight (MW) is often accompanied by limited water solubility and cell permeability, resulting the low bioavailability. RIBOTACs and multivalent binding molecules are also faced with druggability-deficiency due to their generally large MW. Simultaneously, covalent inhibitors have potential toxicity and side effects caused by off-targeting.

Considering the high variability of virus and the complexity of pathogenic mechanisms, DNA-encoded chemistry technology [39], genome editing technology [40], nucleic acid aptamer technology [41] and ligand discovery based on protein self-assembly [42] are expected to provide references for the development of potent antiviral drugs. Moreover, natural products have complex structures and diverse physiological activities, providing unlimited resources to discover novel antiviral drugs [43,44].

The epidemic of COVID-19 reminds the researchers of the urgent demand to develop innovative drugs with broad-spectrum antiviral activities to effectively control the public health crisis and strengthen the technical reserves to combat emerging viral diseases [9]. As an outgrowth of molecular and structural biology, the common mechanisms in different viruses have been gradually revealed, such as the RNA capping machinery [45], the ubiquitous hydrophobic protein structure (viroporins [46]) and host proteins involved in viral infection [47], which will offer novel considerable targets for discovering broad-spectrum antiviral drugs.

Additionally, combining medicinal chemistry with bioinformatics and artificial intelligence technology, high-throughput phenotypic screening and reverse pharmacophore matching virtual screening can performed for listed drugs to explore new drug indications. Furthermore, the establishment of rapid drug screening systems together with reliable safety and effectiveness evaluation systems will also accelerate the development process of antiviral drugs.

Lastly, it should be emphasized that future directions and perspectives on antiviral drug discovery and associated challenges have been discussed by some pioneers in this field, as exemplified by a series of high-quality reviews of Professor Erik De Clercq [1,2,3,48,49,50,51,52,53,54,55,56,57]. Undoubtedly, our endeavors and most achievements in antiviral drug research field have continuously benefited from the inspiration of these articles and his direct guidance. Our long-term close cooperation with Professor Erik De Clercq culminated in the discovery of several antiviral drug candidates for further preclinical studies or clinical trials [58,59,60,61,62,63,64,65,66,67,68]. On the occasion of his 80th anniversary, on behalf of our whole research group, we would like to extend our sincere gratitude and best wishes to Professor Erik De Clercq. 

Collectively, the pioneers of antiviral drugs represented by Professor Erik De Clercq are the models we best learn from. We envision that the extensive application of practical and innovative drug design strategies, as well as integrated screening methods, will facilitate development of novel antiviral therapeutics to counter the existing viral infections, newly emerging infections and the outbreak of new viruses in the future.

## Figures and Tables

**Table 1:** IC_50_ values for NA enzyme inhibition and EC_50_ values for the inhibition of viral replication of compounds ZNV and ZNV–cholesterol conjugate (**12**) [38].

	ZNV		ZNV–Cholesterol Conjugate (12)	
	IC_50_ (nM)	EC_50_ (nM)	IC_50_ (nM)	EC_50_ (nM)
H1N1	0.9 ± 0.4	62.7 ± 4.1	28.0 ± 4.0	36.8 ± 2.1
H3N2	0.7 ± 0.2	87.1 ± 11.9	16.1 ± 8.1	32.1 ± 6.7
H5N1	0.3 ± 0.2	123.4 ± 7.3	24.3 ± 10.1	35.4 ± 10.0
H1N1_H275Y_	1.0 ± 0.2	26.6 ± 6.3	22.0 ± 6.4	22.0 ± 6.2
H3N2_E119V_	10.0 ± 0.7	5240.0 ± 996.3	162.4 ± 8.8	421.7 ± 110.0
